# Identifying and Predicting Intentional Self-Harm in Electronic Health Record Clinical Notes: Deep Learning Approach

**DOI:** 10.2196/17784

**Published:** 2020-07-30

**Authors:** Jihad S Obeid, Jennifer Dahne, Sean Christensen, Samuel Howard, Tami Crawford, Lewis J Frey, Tracy Stecker, Brian E Bunnell

**Affiliations:** 1 Medical University of South Carolina Charleston, SC United States; 2 University of South Florida Tampa, FL United States

**Keywords:** machine learning, deep learning, suicide, suicide, attempted, electronic health records, natural language processing

## Abstract

**Background:**

Suicide is an important public health concern in the United States and around the world. There has been significant work examining machine learning approaches to identify and predict intentional self-harm and suicide using existing data sets. With recent advances in computing, deep learning applications in health care are gaining momentum.

**Objective:**

This study aimed to leverage the information in clinical notes using deep neural networks (DNNs) to (1) improve the identification of patients treated for intentional self-harm and (2) predict future self-harm events.

**Methods:**

We extracted clinical text notes from electronic health records (EHRs) of 835 patients with International Classification of Diseases (ICD) codes for intentional self-harm and 1670 matched controls who never had any intentional self-harm ICD codes. The data were divided into training and holdout test sets. We tested a number of algorithms on clinical notes associated with the intentional self-harm codes using the training set, including several traditional bag-of-words–based models and 2 DNN models: a convolutional neural network (CNN) and a long short-term memory model. We also evaluated the predictive performance of the DNNs on a subset of patients who had clinical notes 1 to 6 months before the first intentional self-harm event. Finally, we evaluated the impact of a pretrained model using Word2vec (W2V) on performance.

**Results:**

The area under the receiver operating characteristic curve (AUC) for the CNN on the phenotyping task, that is, the detection of intentional self-harm in clinical notes concurrent with the events was 0.999, with an F1 score of 0.985. In the predictive task, the CNN achieved the highest performance with an AUC of 0.882 and an F1 score of 0.769. Although pretraining with W2V shortened the DNN training time, it did not improve performance.

**Conclusions:**

The strong performance on the first task, namely, phenotyping based on clinical notes, suggests that such models could be used effectively for surveillance of intentional self-harm in clinical text in an EHR. The modest performance on the predictive task notwithstanding, the results using DNN models on clinical text alone are competitive with other reports in the literature using risk factors from structured EHR data.

## Introduction

### Background and Significance

Suicide ranks among the leading causes of death in the United States. On average, over 100 individuals die of suicide each day, resulting in combined medical and work loss costs totaling approximately US $80 billion annually [1,2]. Numerous risk factors for suicide have been identified and thoroughly researched. For example, suicide is more common in males, American Indian and Alaska Natives, and non-Hispanics and individuals with mental illness (eg, depression, anxiety, substance abuse), previous trauma, communication difficulties, decision-making impulsivity, and aggression [3,4]. Individuals who have previously engaged in intentional self-harm behaviors or suicide attempts are also at increased risk [5,6]. Despite extensive research on various risk factors, prospective suicide prediction remains difficult, as conventionally studied risk factors predict suicide attempts only 26% of the time [5].

Currently established guidelines for suicide risk assessment include clinical interviews and questionnaires administered by qualified health care providers [7,8]. However, research suggests that these approaches exhibit suboptimal performance in predicting future intentional self-harm behavior or suicide [9-11]. Less than a third of patients who engage in intentional self-harm and attempt suicide disclose thoughts about doing so [12]. As such, current methods for identification of at-risk patients can be difficult and time-consuming. A great deal of recent research has focused on addressing these limitations using advanced analytical tools such as natural language processing (NLP) and machine learning [13]. Studies using NLP approaches have largely used electronic health record (EHR)-based [14-16] and NLP- and linguistics-driven prediction models [12,17-19]. Studies using machine learning to predict suicidal and intentional self-harm behaviors from EHR data for patients admitted to hospitals or emergency departments have demonstrated variable accuracy (eg, 65%-95%) [20-24].

Clinical text classification using a deep convolutional network has been useful in the identification of specific phenotypes within the EHR for patients with a given set of clinical signs and symptoms [25,26]. There have been significant advances in recent years in deep learning approaches, such as convolutional neural networks (CNNs), for a variety of applications including text processing and classification, computer vision, and speech recognition [27]. In the area of text processing, there has been significant research in language models that are pretrained and then used to aid in automated text understanding of unlabeled data [28,29]. These resulting learned word vectors could, in turn, be used for clinical text classification tasks [25,26,30]. Pretraining models using these methods provide syntactic and semantic word similarities expressed in a multidimensional vector space with the potential for improving classifications based on neural networks and reducing computational cost [28]. The use of advanced analytical approaches such as deep learning can extend this work and provide distinct advantages in predicting future intentional self-harm, suicide attempts, and suicide.

### Objectives

Deep learning approaches have been used to address topics related to suicide using publicly available data sets. For example, Shing et al [31] compared different machine learning methods including support vector machines (SVM) and a CNN-based model for the assessment of suicide risk based on web-based postings. Although they demonstrated the utility of deep learning, for this specific use case, the SVM model outperformed the CNN model. Conversely, Du et al [32] demonstrated the superiority of a deep learning model over traditional models, including an SVM, in identifying suicide-related tweets in social media data. Despite these examples, there have been no reports in the literature on the utility of deep learning approaches for the identification of suicide-related clinical records (eg, for surveillance purposes) or for the prediction of suicidal behavior using clinical text from an EHR. Improving the recall and precision of phenotyping and predictive algorithms, particularly through deep learning analytic techniques, could lead to better follow-up and care by clinicians for patients who are at risk for intentional self-harm, suicide attempts, suicide, or any combination thereof. In this study, we explored a deep learning approach for (1) the automated detection of intentional self-harm events in clinical text concurrent with International Classification of Diseases (ICD) codes for intentional self-harm, that is, phenotyping and (2) the prediction of future suicide attempts or intentional self-harm based on ICD-labeled encounters within the EHR.

## Methods

### Software Used

We used R version 3.6.1 (R Foundation for Statistical Computing) [33] for processing the data and clinical text and constructing the machine learning pipelines and Keras and TensorFlow v1.13 (Google’s open-source deep neural network framework) for the deep learning models.

### Patient Population

This study was approved by the institutional review board (IRB) for human research at the Medical University of South Carolina (MUSC) under protocol number Pro00087416. Clinical notes were extracted from the Epic (Epic Systems Corporation) EHR system [34] using the MUSC research data warehouse (RDW), which serves as an EHR data repository for research projects. Researchers may request data from the RDW with appropriate IRB approval and data governance oversight [35]. We extracted clinical text notes for adult patients aged 20 to 90 years with ICD codes for suicide attempts or intentional self-harm as defined in the National Health Statistics Report (NHSR) from the Centers for Disease Control and Prevention (CDC) in the United States [36]. The NHSR specifically included codes for self-harm events that were intentional (eg, T42.4X2; poisoning by benzodiazepines, intentional self-harm) and did not include codes for self-harm events that were unintentional (eg, T42.4X1; poisoning by benzodiazepines, accidental). For each patient in the study group, we selected the first intentional self-harm recorded in the chart during the study period (ie, 2012-2019). We filtered the notes within a 24-hour period of the intentional self-harm time stamp. We also extracted clinical text notes for control cases who never had any intentional self-harm ICD codes within our EHR spanning the years 2012 to 2019. The controls were selected randomly from the RDW after matching by age, gender, race, and ethnicity. During the processing of the clinical notes, we matched the controls to the study cases based on the proportion of note types in their records (eg, percent of progress notes) and word length of notes. The matching was performed using the nearest neighbor method in the MatchIt package in R [37]. The resulting patient population included 835 intentional self-harm cases and 1670 controls.

### Clinical Notes

#### Notes Concurrent With Intentional Self-Harm

In the first part of this study, we sought to automate the detection of concurrent intentional self-harm ICD code assignment based on clinical text. The notes included a variety of different note types; however, the majority consisted of progress notes, plan of care notes, emergency department (ED) provider notes, history and physical (H&P) notes, and consult notes. A full list of note types and their relative frequencies is provided in a table in Multimedia Appendix 1. Individual notes longer than 800 words (less than one-third of all notes) were truncated at 800. We chose this cutoff to include as many notes per patient as possible. Notes belonging to the same patient were then concatenated into a single string arranged temporally, yielding 1 record per patient. Concatenated strings longer than 8000 words (44/2505, 1.76% of patients) were truncated at 8000. This allowed us to maintain the generated token vectors within a reasonable range for computational performance. The patients were divided into a training and cross-validation set (2012-2017) with 661 intentional self-harm cases and 1502 controls and a holdout test set (2018-2019) with 174 intentional self-harm cases and 168 controls.

#### Prediction From Previous Clinical Notes

In this part of the study, we sought to predict the future occurrence of intentional self-harm events based on previous clinical notes within the EHR. Clinical text was collected from a predictive window for a period between 180 days to 30 days before the index event (ie, the first reported intentional self-harm event on record) for each patient. Patients who did not have clinical notes during that time window were excluded. Clinical notes were used from the first date within that time window up to 90 days following the first date or up to 30 days before the intentional self-harm event (whichever is first). That is, the largest possible predictive window included clinical notes from a time interval of up to 90 days. The same time window was used for the control group; however, the latest visit on record within the study period was used as the index visit instead of an intentional self-harm event. To reduce noise and excessive amounts of notes in this part of the study, we limited notes to the following note types: progress notes, ED provider notes, H&P notes, consult notes, and discharge summaries. Individual notes were truncated to 1500 words and concatenated texts to 10,000-word cutoffs to capture a wider set of clinical texts. For the prediction part of the study, the patients were divided into a training and cross-validation set (2012-2017) with 480 intentional self-harm cases and 645 controls and a holdout test set (2018-2019) with 106 intentional self-harm cases and 106 controls.

### Labeling the Test Set

A sample of 200 records from the test set (2018-2019) was manually reviewed to provide gold standard labels for a comparison with ICD code labels (based on the NHSR from the CDC). Each record reflected clinical notes in the EHR from concurrent visits of patients. We selected a random 100 from the study group (with intentional self-harm ICDs) and 100 controls. The concatenated strings from concurrent notes for this sample were imported into REDCap (Research Electronic Data Capture) [38] and made available for review and labeling by the reviewers on our research team, which included 3 clinical psychologists, a psychiatry resident, a medical student, and a pediatrician. The reviewers were instructed to label the notes as intentional self-harm if there was a suicide attempt or intentional self-harm noted in any of the clinical notes associated with the concurrent visit. Suicidal ideation alone was not considered intentional self-harm. A subsample of 100 notes was labeled independently by 2 labelers to estimate the interrater reliability.

### Text Processing

We tested several machine learning algorithms using the training data, including both deep learning–based classifiers using word embeddings (WEs) and the traditional bag-of-words (BOW)–based models. We performed the necessary preprocessing of the text for both types. We used the quanteda R package [39] and regular expression functions within R for the text-processing pipeline. For the traditional BOW models, text processing included lower casing; removal of punctuation, stop words, and numbers; word stemming; and tokenization. For the WE models, text processing included lower casing, sentence segmentation, removal of punctuation, replacement of large numbers and dates with tokens using regular expressions, and tokenization.

### Word Frequencies

Before running the machine learning algorithms, we examined differences in word frequencies across clinical notes concurrent with intentional self-harm events and notes preceding intentional self-harm events by over 30 days as compared with clinical notes from the control population. We performed a chi-square analysis to assess keywords that are overrepresented across the corpora of text [40].

### Bag-of-Words−Based Classifiers

For the BOW models, word frequencies were used as features and were normalized using term frequency–inverse document frequency [41]. The traditional text classification models included naïve Bayes [42]; decision tree classifier [43] with a maximum depth of 20; random forest (RF) [44] with 201 trees and the number of variables randomly sampled as candidates at each split (mtry=150); SVM [45] type 1 with a radial basis kernel [46]; and a simple multilayer perceptron (MLP) artificial neural network with a 64-node input layer, a 64-node hidden layer, and a single output node. We used the rectified linear unit (ReLU) activation function in both the input and hidden layers and sigmoid activation for the binary output node. The MLP was trained using a learning rate of 1×10^−4^, a batch size of 32, and a 20% validation split over 30 epochs.

### Word Embeddings

We used Keras [47] and TensorFlow version 1.13 [48] for constructing and training the deep learning models. In preparation for WE, the text strings were converted to token sequences. To construct the features for the deep learning models, the sequences were prepadded with zeros to match the length of the longest string in the training set. We used Word2vec (W2V) to generate a pretrained model [28]. The W2V weights were derived by pretraining a W2V skip-gram model on a sample of over 800,000 clinical notes from our EHR data set using 200 dimensions per word, a skip window size of 5 words in each direction, and negative sampling of 5. To explore and visualize the outcome of the pretrained W2V model, we used the t-distributed stochastic neighbor embedding (t-SNE) to map the multidimensional word vectors into a 2D space [49]. The performance of each deep learning classifier was assessed with either randomly initialized embeddings or W2V-initialized embeddings.

### Deep Learning Models

We examined 2 different deep neural network (DNN) architectures: a CNN architecture similar to a previously published model [26] and a long short-term memory (LSTM) model [50]. Both architectures were tested using either randomly initialized WE weights in Keras or WE initialized with the weights from the pretrained W2V.

Both models had WE with 200 dimensions per word. The input layer had a dimension size slightly exceeding the maximum length of the input sequences of tokens, which were 8352 tokens for the concurrent notes and 11,000 tokens for the predictive notes. The CNN architecture consisted of an input layer; a WE layer included with a drop rate of 0.2; a convolutional layer with multiple filter sizes (3, 4, and 5) in parallel, with 200 nodes in each, ReLU activation, a stride of one, and global max-pooling; a merge tensor then a fully connected 200-node hidden layer with ReLU activation and a drop rate of 0.2; and an output layer with a single binary node with a sigmoid activation function. The LSTM architecture consisted of an input layer; a WE layer with a drop rate of 0.1; an LSTM layer with 64 nodes; both global average pooling and global max-pooling layers with a merge tensor of the 2; a fully connected 100-node hidden layer with ReLU activation and a drop rate of 0.1; and a single sigmoid binary output node.

The DNN models were trained using an adaptive moment estimation gradient descent algorithm [51] with a diminishing learning rate starting at 4×10^−4^, batch size of 32, validation split at 15%, and early stopping based on the loss function for the validation data with patience of 5.

### Training and Evaluation

#### Detection of Concurrent Intentional Self-Harm

For the automated detection of concurrent intentional self-harm ICD code assignment based on clinical text, we used the training and cross-validation data set (with index visits from 2012-2017) to identify the best performing models and hyperparameters. We then used the top 2 performing models (the DNNs) for training on the full training set and testing on the holdout test set (with index visits from 2018 to 2019), which included the 200 manually reviewed cases. The models were trained using intentional self-harm ICD codes as positive labels. However, we tested the output using both intentional self-harm ICD codes as positive labels and manually reviewed (gold standard) labels.

#### Prediction of Future Intentional Self-Harm Events

The 2 best performing models, namely, the DNNs, were used to predict future intentional self-harm events based on previous clinical notes. In the holdout test set, we used a balanced set with an equal number of intentional self-harm cases and controls with 106 cases in each. The DNN models were trained on notes preceding the first intentional self-harm visits during the 2012 to 2017 time frame and then tested on notes preceding the first intentional self-harm visits during the 2018 to 2019 time frame. Unlike the previous task, which had near-ceiling performance results with little variation, the performance of the DNNs on the predictive task varied between different runs of the same model even when using the same training and testing sets. This is due to the random initialization of weights in TensorFlow and random shuffling between epochs during training. To evaluate the performance of the different DNN architectures more precisely, we ran each model 50 times and examined the averages of the different metrics and used the Student *t* test (two-tailed) to determine statistical differences in performance.

#### Metrics

The performance metrics for all experiments, including area under the receiver operating characteristic (ROC) curve (AUC), were calculated in R using the caret [52] and pROC [53] packages. We also calculated the accuracy, precision, recall, and F1 score for all the models.

## Results

### International Classification of Diseases Code Analysis

The interrater reliability during the manual review exhibited a Cohen kappa of 0.96. Using the labels from the manual review as the gold standard, the accuracy of the intentional self-harm ICD codes attributed to concurrent visits was 0.92, with a precision of 0.84 and recall of 1.0. Thus, 16 cases out of 100 that were assigned an intentional self-harm ICD code did not exhibit intentional self-harm as part of the presenting history, per the manual review. However, all but 2 of the 16 *false-positives* by ICD had past intentional self-harm mentioned in their clinical notes. For those 2, 1 was suspected intentional self-harm, and the other had a previous admission for suicidal ideation with possible intentional self-harm.

### Word Frequency Results

The result from this analysis overrepresented keywords in clinical notes concurrent with intentional self-harm events and clinical notes before the intentional self-harm events (Table 1). For example, the words *suicide* and *attempt* top the list in concurrent notes; however, they do not rank in the top 10 words in preceding notes. Instead, the words *disorder* and *si* (the shorthand for suicidal ideation) top the list in notes preceding intentional self-harm.

**Table 1:** The top 10 words in each group were compared with controls, along with the chi-square statistic for each.

Concurrent with ISH^a,b^		Before ISH^c^	
Keyword	Chi-square (*df*=1)	Keyword	Chi-square (*df*=1)
suicide	1.3E+5	disorder	1.2E+4
attempt	8.2E+4	si^d^	8.5E+3
overdose	6.7E+4	suicidal	6.0E+3
si	6.5E+4	mood	5.8E+3
disorder	5.2E+4	use	4.7E+3
suicidal	5.2E+4	alcohol	4.6E+3
psychiatry	4.0E+4	qhs^e^	4.5E+3
iop^f^	3.6E+4	safety	4.2E+3
interview	3.5E+4	interview	3.9E+3
mood	2.9E+4	cocaine	3.9E+3

^a^Keywords from clinical notes from visits concurrent with ISH events.

^b^ISH: intentional self-harm.

^c^Keywords from clinical notes from visits before the first ISH events.

^d^si: suicidal ideation.

^e^iop: Institute of Psychiatry.

^f^qhs: every bedtime (from Latin quaque hora somni).

### Word2vec Pretraining Results

**Figure 1:**
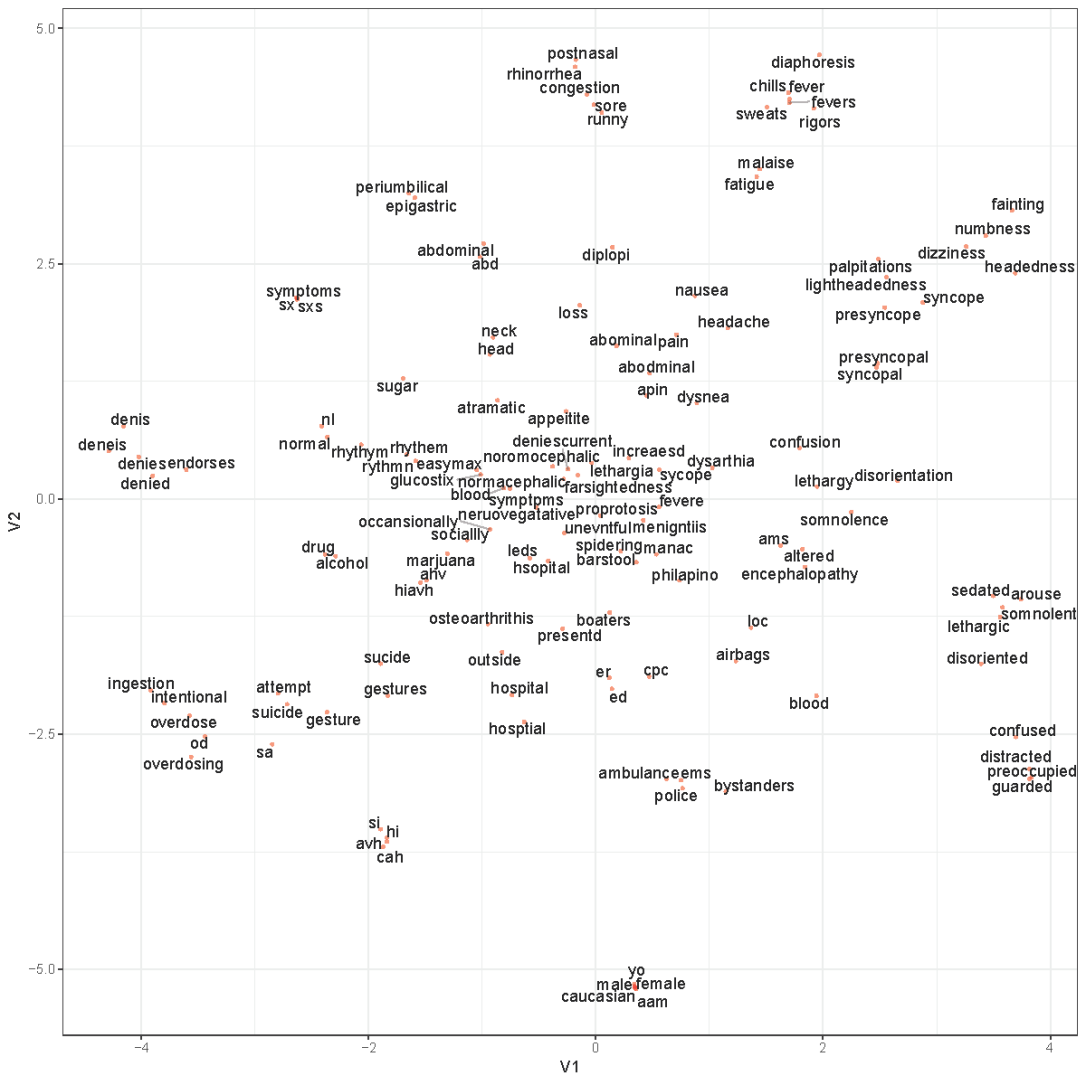
A visualization of a sample of relevant words derived from the Word2vec model reduced into two dimensions using t-distributed stochastic neighbor embedding. V1=variable 1; V2=variable 2.

The W2V model successfully clustered words that seemed to have similar semantic contexts. Figure 1 shows the visualization of a sample of relevant words reduced into 2 dimensions using the t-SNE algorithm. Table 2 shows the top 10 words semantically similar to *attempt* and the top 10 words similar to *ideation* along with their cosine similarities. For example, the cosine similarity between *attempt* and *suicide* WE vectors was 0.730 and between *ideation* and *suicidal* was 0.872. The list also shows several misspelled words in a similar dimension space as their correctly spelled counterparts.

**Table 2:** Words semantically similar to the words attempt and ideation and their cosine similarity in the 200-dimension vector space as identified by the Word2vec analysis.

Term		Cos sim^a^
*attempt*		
	attempt	1.000
	suicide	0.730
	overdose	0.696
	osteoarthrithis	0.679
	gesture	0.643
	sucicide	0.625
	benzodiaspines	0.619
	intentional	0.617
*ideation*		Cos sim^a^
	ideation	1.000
	suicidal	0.872
	homicidal	0.837
	ideations	0.736
	intent	0.681
	ideaiton	0.651
	si^b^	0.648
	sucidial	0.619

^a^Cos sim: cosine similarity.

^b^si: suicidal ideation.

### Detection of Concurrent Intentional Self-Harm

#### Training and Cross-Validation

Table 3 shows the results of the automated detection of concurrent intentional self-harm ICD code assignment based on the training and cross-validation data set with intentional self-harm visits during the period of 2012 to 2017. The DNNs outperformed the BOW classifiers. The CNN models had the highest AUC and F1 score. The best performance overall was for the CNN with W2V WE (CNNw) with an AUC of 0.988 and an F1 score of 0.928. The CNN with randomly initialized WE (CNNr) was a close second, with significantly overlapping 95% CIs. The LSTMs with randomly initialized WE (LSTMr) and the LSTM with W2V WE (LSTMw) AUCs were 0.982 and 0.975, respectively, with F1 scores above 0.887.

Among the BOW models, RF had the best AUC (0.961), and MLP had the best F1 score (0.862). On the basis of these results, we used 2 deep learning models for the rest of this study.

**Table 3:** The metrics for training and cross-validation on the 2012 to 2017 data set.

Model	AUC^a^ (95% CI^b^)	Accuracy (95% CI)	Precision	Recall	F1 score
NB^c^	0.908 (0.882-0.934)	0.870 (0.839-0.898)	0.734	0.865	0.794
DT^d^	0.870 (0.839-0.901)	0.865 (0.833-0.893)	0.715	0.885	0.791
RF^e^	0.961 (0.944-0.978)	0.896 (0.867-0.921)	0.794	0.865	0.828
SVM^f^	0.947 (0.925-0.969)	0.900 (0.872-0.924)	0.859	0.782	0.819
MLP^g^	0.957 (0.938-0.976)	0.917 (0.890-0.939)	0.828	0.897	0.862
CNNr^h^	0.984 (0.972-0.995)	0.946 (0.924-0.964)	0.938	0.872	0.904
CNNw^i^	0.988 (0.977-0.999)	0.959 (0.939-0.974)	0.947	0.910	0.928
LSTMr^j^	0.982 (0.972-0.992)	0.943 (0.920-0.961)	0.919	0.878	0.898
LSTMw^k^	0.975 (0.960-0.990)	0.937 (0.913-0.956)	0.918	0.859	0.887

^a^AUC: area under the receiver operating characteristic curve.

^b^CI: 95% confidence intervals for the AUC.

^c^NB: naïve Bayes.

^d^DT: decision tree.

^e^RF: random forest.

^f^SVM: support vector machine.

^g^MLP: multilayer perceptron.

^h^CNNr: convolutional neural network with randomly initialized word embeddings.

^i^CNNw: convolutional neural network with Word2vec word embeddings.

^j^LSTMr: long short-term memory with randomly initialized word embeddings.

^k^LSTMw: long short-term memory with Word2vec word embeddings.

#### Testing of Concurrent Intentional Self-Harm Labels

**Figure 2:**
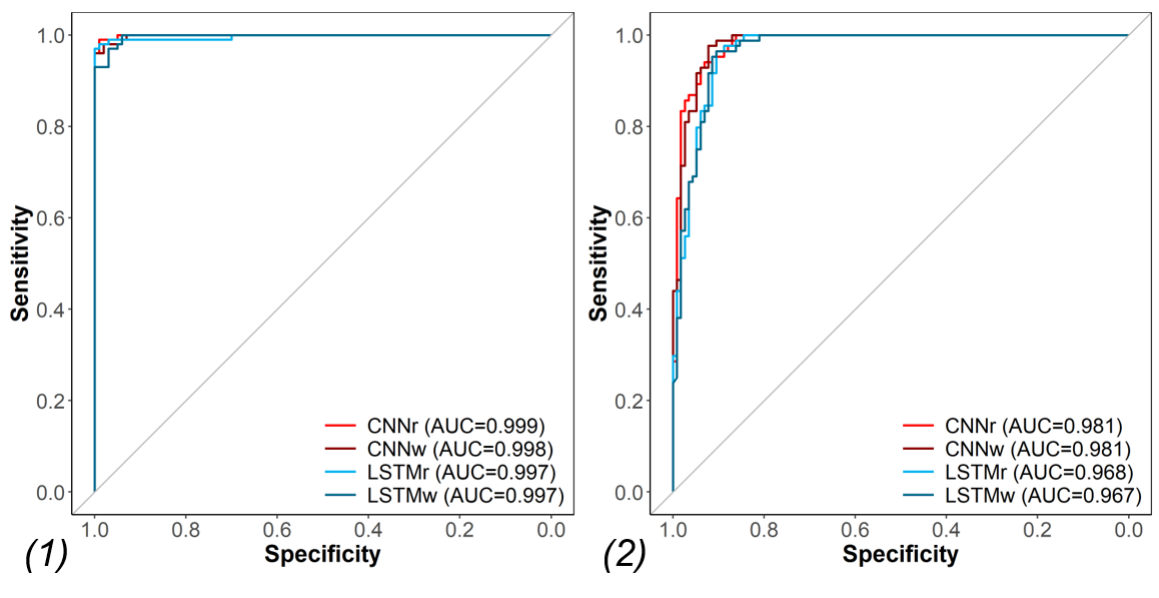
The area under the receiver operating characteristic curve for training on the 2012 to 2017 data set and testing on the holdout test set (2018-2019) using (1) International Classification of Diseases labels and (2) gold standard labels. AUC: area under the receiver operating characteristic curve; ICD: International Classification of Diseases; CNNr: convolutional neural network with randomly initialized word embeddings; CNNw: convolutional neural network with Word2vec word embeddings; LSTMr: long short-term memory with randomly initialized word embedding; LSTMw: long short-term memory with Word2vec word embedding.

Training the models on the full 2012 to 2017 data set then testing on the holdout (2018-2019) test set yielded even better performance than in the above cross-validation for detecting concurrent intentional self-harm ICD labels (Table 4). The best performing model was the CNNr with an AUC of 0.999 and an F1 score of 0.985. A plot of the training history for this task shows that the model converges smoothly to a minimum loss value on both training and validation (Multimedia Appendix 2). There was no advantage to adding the pretrained W2V WE, that is, the CNNw when testing on the holdout set. The CNNs slightly outperformed the LSTMs, but the results in all models were close to ceiling, making it difficult to point out the significance of these differences. As expected, as the models were trained on ICD labels, they performed better in predicting concurrent ICD labels than they did with predicting the gold standard labels (Figure 2). Of note, is that the recall remained very high when testing on the gold standard labels compared with the ICD labels, whereas the precision suffered slightly reflecting the precision achieved during the intentional self-harm ICD code analysis.

**Table 4:** The metrics for training on the 2012 to 2017 data set and testing on the 2018 to 2019 holdout test set using both International Classification of Diseases labels and gold standard labels.

Model			AUC^a^ (95% CI^b^)		Accuracy (95% CI)		Precision		Recall		F1 score
**ICD^c^labels**											
	CNNr^d^	0.999 (0.998-1.000)		0.985 (0.957-0.997)		0.980		0.990		0.985	
	CNNw^e^	0.998 (0.996-1.000)		0.970 (0.936-0.989)		0.980		0.960		0.970	
	LSTMr^f^	0.997 (0.991-1.000)		0.980 (0.950-0.995)		0.990^d^		0.970		0.980	
	LSTMw^g^	0.997 (0.994-1.000)		0.960 (0.923-0.983)		0.989		0.930		0.959	
**Gold standard labels**											
	CNNr^c^	0.981 (0.966-0.997)		0.915 (0.867-0.950)		0.832		1.000		0.908	
	CNNw^e^	0.981 (0.965-0.997)		0.920 (0.873-0.954)		0.847		0.988		0.912	
	LSTMr^f^	0.968 (0.946-0.989)		0.910 (0.861-0.946)		0.837		0.976		0.901	
	LSTMw^g^	0.967 (0.945-0.989)		0.920 (0.873-0.954)		0.862		0.964		0.910	

^a^AUC: area under the receiver operating characteristic curve.

^b^CI: 95% confidence intervals for the AUC.

^c^ICD: International Classification of Diseases.

^d^CNNr: convolutional neural network with randomly initialized word embeddings.

^e^CNNw: convolutional neural network with Word2vec word embeddings.

^f^LSTMr: long short-term memory with randomly initialized word embeddings.

^g^LSTMw: long short-term memory with Word2vec word embeddings.

### Prediction of Future Intentional Self-Harm Events

**Figure 3:**
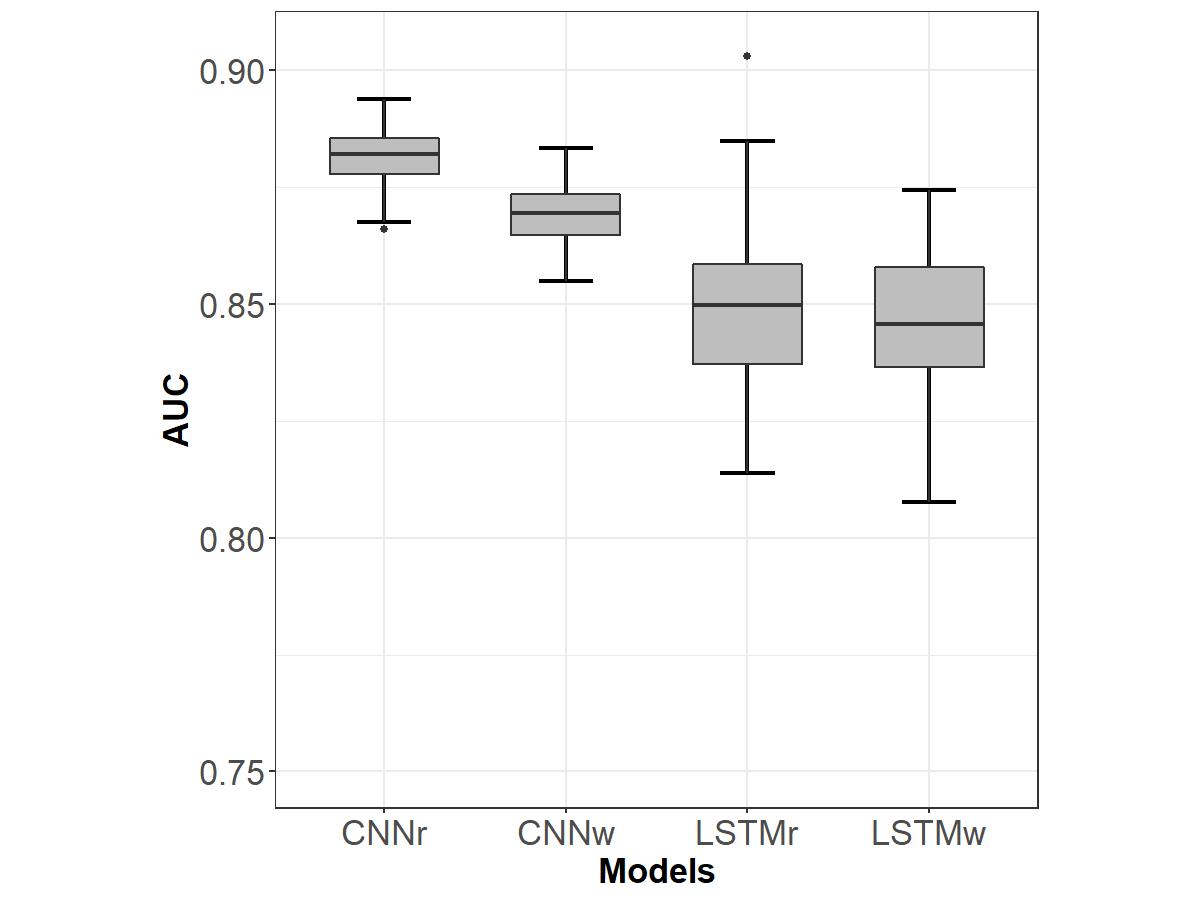
The mean area under the receiver operating characteristic curve and 95% CI for models trained on notes preceding the first intentional self-harm visits in patients presenting during the 2012 to 2017 time frame and tested on notes preceding the first intentional self-harm visits in patients presenting during the 2018 to 2019 time frame. The differences in performance were all significant (*P*<.001) except for the difference between the LSTMr and LSTMw. AUC: area under the receiver operating characteristic curve; CNNr: convolutional neural network with randomly initialized word embeddings; CNNw: convolutional neural network with Word2vec word embeddings; LSTMr: long short-term memory with randomly initialized word embedding; LSTMw: long short-term memory with Word2vec word embedding.

The results for the prediction of future intentional self-harm events based on previous clinical notes are shown in Table 5. These values are the means of the different metrics after 50 training and testing cycles for each model. Figure 3 shows the differences in performance between the different models. The CNNr model had the best performance, with a mean AUC of 0.882 and a standard deviation of 0.006 (*P*<.001) compared with CNNw, which in turn outperformed the LSTM models (*P*<.001). There was no significant difference between LSTMr and LSTMw. The variance in performance was notably wider in the LSTM models than in the CNN models. Multimedia Appendix 3 shows the ROC curves for each of the models highlighting the mean AUC. Although pretraining with W2V did not add value in terms of performance, it did reduce the number of epochs needed during training by an average of 32% for the CNN and 12% for the LSTM.

**Table 5:** The metrics for models trained on notes preceding the first intentional self-harm visits in patients presenting during the 2012 to 2017 time frame and tested on notes preceding the first intentional self-harm visits in patients presenting during the 2018 to 2019 time frame.

Model	AUC^a^ (95% CI^b^)	Accuracy (95% CI)	Precision	Recall	F1 score
CNNr^c^	0.882 (0.871-0.891)	0.792 (0.774-0.807)	0.863	0.694	0.769
CNNw^d^	0.869 (0.858-0.879)	0.782 (0.766-0.792)	0.860	0.673	0.755
LSTMr^e^	0.850 (0.827-0.877)	0.758 (0.729-0.788)	0.830	0.656	0.729
LSTMw^f^	0.846 (0.819-0.871)	0.750 (0.717-0.778)	0.822	0.644	0.720

^a^AUC: area under the receiver operating characteristic curve.

^b^CI: 95% confidence intervals for the AUC.

^b^CNNr: convolutional neural network with randomly initialized word embeddings.

^d^CNNw: convolutional neural network with Word2vec word embeddings.

^e^LSTMr: long short-term memory with randomly initialized word embeddings.

^f^LSTMw: long short-term memory with Word2vec word embeddings.

## Discussion

### Semantic Differences

The word frequency analyses identified keywords that were overrepresented in clinical notes associated with intentional self-harm visits. As noted in Table 1, words such as *attempt* and *overdose* were highly overrepresented in clinical notes concurrent with intentional self-harm events compared with controls. Conversely, suicidal ideation (as represented by the shorthand word *si*) was frequently present in preintentional self-harm notes. This is consistent with the literature on ideation, which is a prominent risk factor for suicide attempts and completions [54].

The W2V pretraining on our full data set of clinical notes successfully clustered relevant words together. It also demonstrated word similarity for some of the significant words identified above. For example, the words *attempt*, *suicide*, and *overdose* were closely linked with high cosine similarity. This model was also useful in clustering misspelled words with their correctly spelled counterparts, which may help reduce noise due to misspelling in the clinical notes.

### Detection of Intentional Self-Harm Events

The deep learning models outperformed BOW models in identifying intentional self-harm in training and testing using the 2012 to 2017 data set. Given this outcome, we trained the deep learning models on the full 2012 to 2017 data set and then used the 2018 to 2019 data set as a holdout test set. This temporal division of the data is intended to replicate a real-world scenario where models could be trained on historical data to identify intentional self-harm in new records. The results show that we can accurately detect intentional self-harm events in concurrent clinical notes with intentional self-harm ICD codes. More specifically, we showed that a model trained on aggregated clinical text associated with a given intentional self-harm visit may be used to identify concurrent intentional self-harm events even if ICD codes were not yet provided or assigned. In other words, clinical text alone is useful in accurately identifying the intentional self-harm phenotype.

Although there is limited literature on the performance of NLP and machine learning approaches for the phenotyping of intentional self-harm, our DNN classifiers with precisions up to 99% for concurrent notes with intentional self-harm ICD codes and up to 86% for gold standard intentional self-harm events compare favorably with previous reports, especially when considering that the models were trained on ICD codes as labels. Using a hybrid machine learning and rule-based NLP approach, Fernandes et al [19] achieved a precision of 82.8% for identifying suicide attempts. Another study comparing the accuracy of ICD codes and NLP-extracted concepts for suicidality achieved a precision of 60% using NLP alone and 97% using both ICD-9 codes and NLP; however, this study did not differentiate between suicidal ideation and intentional self-harm [16].

Although the CNN-based models seemed to slightly outperform the LSTM-based models on the phenotyping task, it is difficult to show a significant advantage to using either model or the advantage of pretraining with W2V due to the near-ceiling performance of all the DNNs on this task and the relatively small data set.

Nonetheless, a DNN model trained using this method may be useful for surveillance purposes and could well supplement surveillance using ICD codes. Training such a model using intentional self-harm ICD codes as positive labels is dependent on reliable assignment of ICD codes. Fortunately, ICD codes for intentional self-harm at our institution were accurate, as shown by the manual review of charts, notwithstanding the limitation of a relatively high false-positive rate. Finally, accurate phenotyping of the intentional self-harm events paves the way for future directions in identifying other phenotypes, for example, those with suicidal ideation alone versus intentional self-harm or not intentional self-harm, which may or may not have accurate ICD codes. Such precise or deep phenotyping is an important step toward predicting the risk of mortality, given the availability of mortality data.

### Prediction of Future Intentional Self-Harm Based on Clinical Text

The results also show that aggregated clinical notes from visits between 1 and 6 months before the index visit predicted future intentional self-harm events with an AUC of 0.882 for the *best performing* CNN model. These results compare favorably with the literature on predictive models for suicide attempts. Using a complex combination of structured EHR data (including demographics, diagnostic codes, and census-based socioeconomic status) and medication data extracted via NLP, Walsh et al [20] achieved a maximum AUC of 0.84. Moreover, this AUC was based only on 7-day-old data. The AUC dropped gradually to 0.81 as the predictive window widened to 6 months before the index visit.

When comparing the performance between the 2 DNN architectures, we noted a consistent and statistically significant performance advantage of the 2 CNN models over the LSTM-based ones (Figure 3). Moreover, the LSTM had a relatively high variance and inconsistent performance over the 50 training runs, as can be noted from the CIs. We also noted a higher computational cost for the LSTM over the CNN (almost twice the time needed for training per epoch). In addition to the higher computational cost, recurrent neural networks show a minor advantage in generic text classification tasks [55,56]. At least with a small data set like ours, the CNNs were found to converge more smoothly and provide better performance.

While the W2V pretraining clustered similar words, initializing the WE layer with W2V weights did not add any value to either of the predictive models. Although CNNr (AUC=0.882) performed only slightly better than CNNw (AUC=0.869), the difference was statistically significant. However, there was no difference between the LSTMr and LSTMw. These results were unexpected given the advantages of pretrained WE in picking up misspellings and word similarities and highlight the need to examine newer, more complex language models such as Google’s (Alphabet Inc) Bidirectional Encoder Representations from Transformers [29].

Regardless of the model architecture, these results are promising. Such predictive models may be useful in stratifying hospitalized patients into risk categories, which may aid in discharge planning. Using technology (telephone, emails, or text messages) for follow-up in the postdischarge period has been shown to reduce risk of future suicide attempts [57]. Furthermore, patients could be prophylactically assigned a social worker; be directed to collaborative primary care clinics with access to mental health services; or receive mental health referrals, telehealth appointments, or home health visits [58]. Adequate refinement of a predictive model may even allow for stratification of patients to a level of care necessary post discharge, beyond simple binary risk categorization.

### Limitations

To identify patients with intentional self-harm during a given visit, we trained the models on ICD codes. Therefore, they can only perform as well as the ICD code designation. As mentioned earlier, during the manual labeling process, several patients had a past medical history of intentional self-harm rather than suicide attempt or self-harm as part of the presenting chief complaint or diagnosis. A possible solution would be to train models to introduce multiple labels that include current and past intentional self-harm through manual review. However, this would require a manual review of several hundreds of charts, which was beyond the scope of this initial pilot work.

Moreover, although we can clearly identify intentional self-harm, this still does not specify *intent to die*. This highlights the need for data on fatalities due to suicide. There are multiple forms of self-injury (eg, firearms, sharp objects, jumping from a high place) with ICD codes that are not accompanied by the classification of intent to harm oneself. Therefore, in these instances of unknown intent, self-injury may reflect a multitude of motives: communicating distress, suicidal gestures with low lethality, nonsuicidal self-injury (NSSI), or fatality [59]. Existing literature predicting NSSI behaviors yields 3 notable risk factor categories: history of NSSI, cluster B personality, and hopelessness [60]. Identifying NSSI can be of a significant prognostic value and has not been distinguished from intent to die in this study.

Another limitation of this study is that our model currently only addresses features within clinical texts. Other clinical information could be added to the model, such as associated demographics, comorbidities, and risk factors (eg, codes for depression or substance use). Moreover, with respect to suicide prediction, EHR data alone may not provide a full picture. Ideally, our data should be linked with the statewide cause of death data, which should yield an improved predictive power.

Although deep learning models are more powerful, they are less interpretable than some of the BOW models. For example, when using an RF model, the results of a variable importance analysis may yield insight into significant words. In fact, it may be beneficial to use both types of predictive models in mental health applications. This would leverage the power of deep learning models as well as the advantages of interpretable models. Future work should also include the exploration of attention-based deep learning models with some insight into explainability [61], which may address the utility of these models in real-world clinical decision support and adoption by clinicians.

Finally, the results presented here are based on data from a single EHR system at 1 academic medical center, making it difficult to draw generalizations about the high level of performance of our models in other environments. Future work should include collaboration with other institutions to ascertain the performance of these models in other environments.

### Conclusions

Most of the models showed relatively good performance when detecting intentional self-harm events in concurrent clinical notes, that is, the phenotyping task. This is likely due to a strong signal within concurrent notes and is associated with a high fidelity of ICD code attribution for intentional self-harm, at least at our institution. When applied to the prediction of a future occurrence of intentional self-harm code assignment in a patient chart based on previous clinical notes, the AUC dropped to 0.882 with a modest recall and precision. Nevertheless, our results are competitive with the results from other models reported in the literature. Improving the precision of these algorithms could lead to better follow-up and preventative care by mental health professionals for patients who are at risk for future suicide attempts.

## Appendix

Multimedia Appendix 1 The full list of note types and their relative frequencies in the data set.

Multimedia Appendix 2 A plot of the convolutional neural network model’s training history for the phenotyping task. The learning curve shows that the model converges smoothly to a minimum loss value on both training and validation sets using an Adam optimizer.

Multimedia Appendix 3 Plots of the receiver operating characteristic curves for the 50 training and testing runs for all the models highlighting the mean area under the receiver operating characteristic curve for each model.

## Abbreviations

AUC: area under the receiver operating characteristic curve
BOW: bag-of-words
CDC: Centers for Disease Control and Prevention
CNN: convolutional neural network
CNNr: CNN with randomly initialized word embedding
CNNw: CNN with Word2vec word embedding
DNN: deep neural network
ED: emergency department
EHR: electronic health record
H&P: history and physical
ICD: International Classification of Diseases
IRB: institutional review board
LSTM: long short-term memory
LSTMr: LSTM with randomly initialized word embedding
LSTMw: LSTM with Word2vec word embedding
MLP: multilayer perceptron
MUSC: Medical University of South Carolina
NHSR: National Health Statistics Report
NLP: natural language processing
NSSI: nonsuicidal self-injury
RDW: research data warehouse
ReLU: rectified linear unit
RF: random forest
ROC: receiver operating characteristic
SVM: support vector machines
t-SNE: t-distributed stochastic neighbor embedding
W2V: Word2vec
WE: word embedding
