# Supplementary material for: Identifying and Predicting Intentional Self-Harm in Electronic Health Record Clinical Notes: Deep Learning Approach
Source: JMIR Med Inform. 2020 Jul 30;8(7):e17784. doi: 10.2196/17784 (PMC7426805; doi:10.2196/17784)
Supplement: Multimedia Appendix 1 [file medinform_v8i7e17784_app1.docx]

**Multimedia Appendix 1**

The full list of note types and their relative frequencies in the data set.

| **Note types** | **n** |
| --- | --- |
| PROGRESS NOTES | 3942 |
| PLAN OF CARE | 2360 |
| ED PROVIDER NOTES | 1565 |
| H&P | 731 |
| CONSULTS | 696 |
| IP-CONSULT | 539 |
| TELEPHONE ENCOUNTER | 301 |
| DISCHARGE SUMMARIES | 256 |
| DISCHARGE INSTR - OTHER ORDERS | 219 |
| IP-H & P | 179 |
| TRANSFER OF CARE | 172 |
| SW/CM | 168 |
| MEDICAL STUDENT | 142 |
| TRAUMA | 113 |
| ASSESSMENT & PLAN NOTE | 72 |
| TREATMENT PLAN | 64 |
| H&P (VIEW-ONLY) | 63 |
| TRANSFER SUMMARY | 51 |
| SMOKING CESSATION | 46 |
| DISCHARGE SUMMARY | 16 |
| IP-DISCHARGE | 10 |
| DEATH SUMMARY | 6 |
| SECLUSION AND RESTRAINT TREATMENT PLAN | 4 |
| DISCHARGE SUMMARY IOP | 3 |
| MEDFLOW PATIENT SUMMARY | 3 |
| ED FOLLOW UP | 2 |
| PATIENT CARE CONFERENCE | 1 |
