# Supplementary figures and images for: Identifying and Predicting Intentional Self-Harm in Electronic Health Record Clinical Notes: Deep Learning Approach
Source: JMIR Med Inform. 2020 Jul 30;8(7):e17784. doi: 10.2196/17784 (PMC7426805; doi:10.2196/17784)

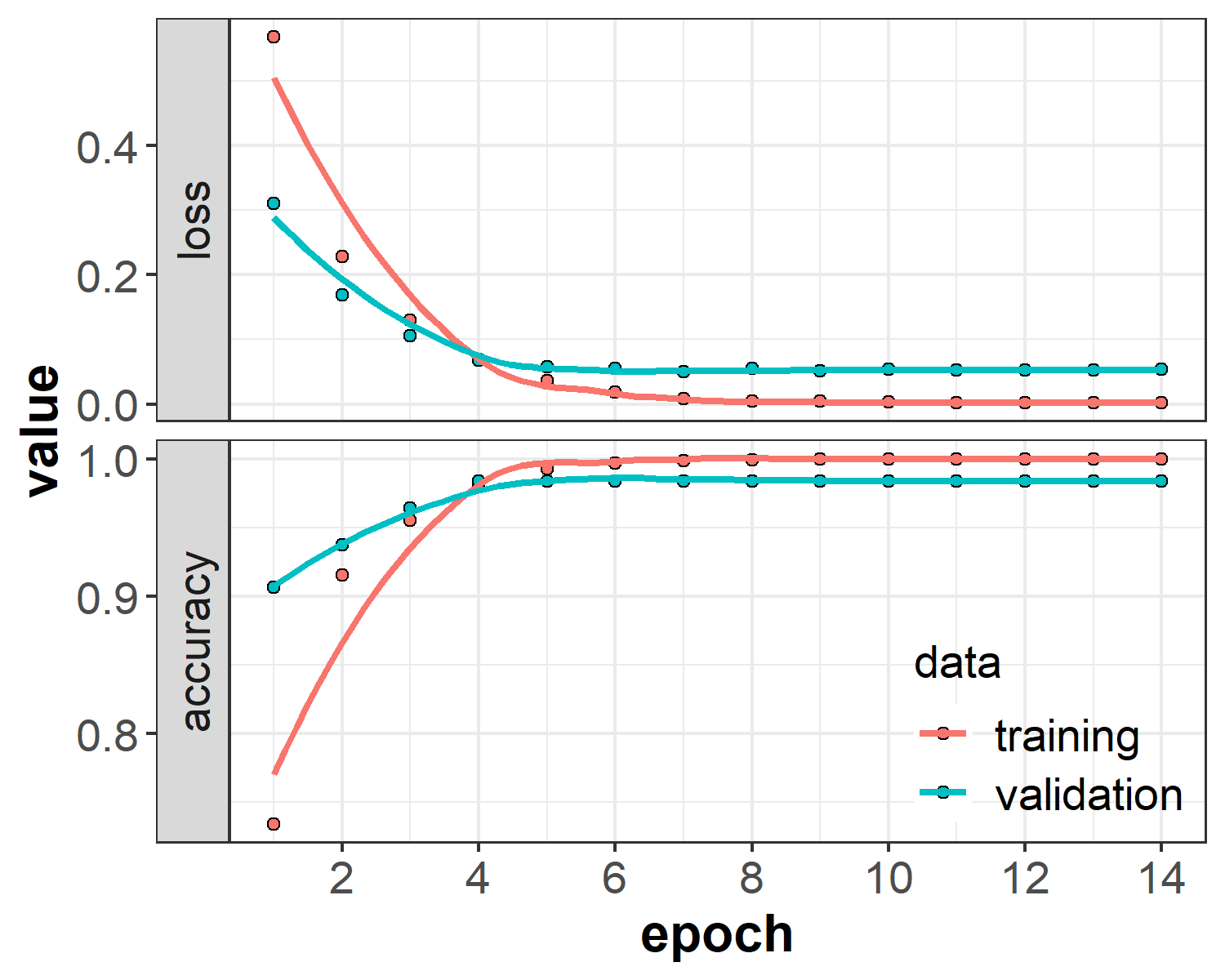

Supplement: Multimedia Appendix 2 [file medinform_v8i7e17784_app2.png]

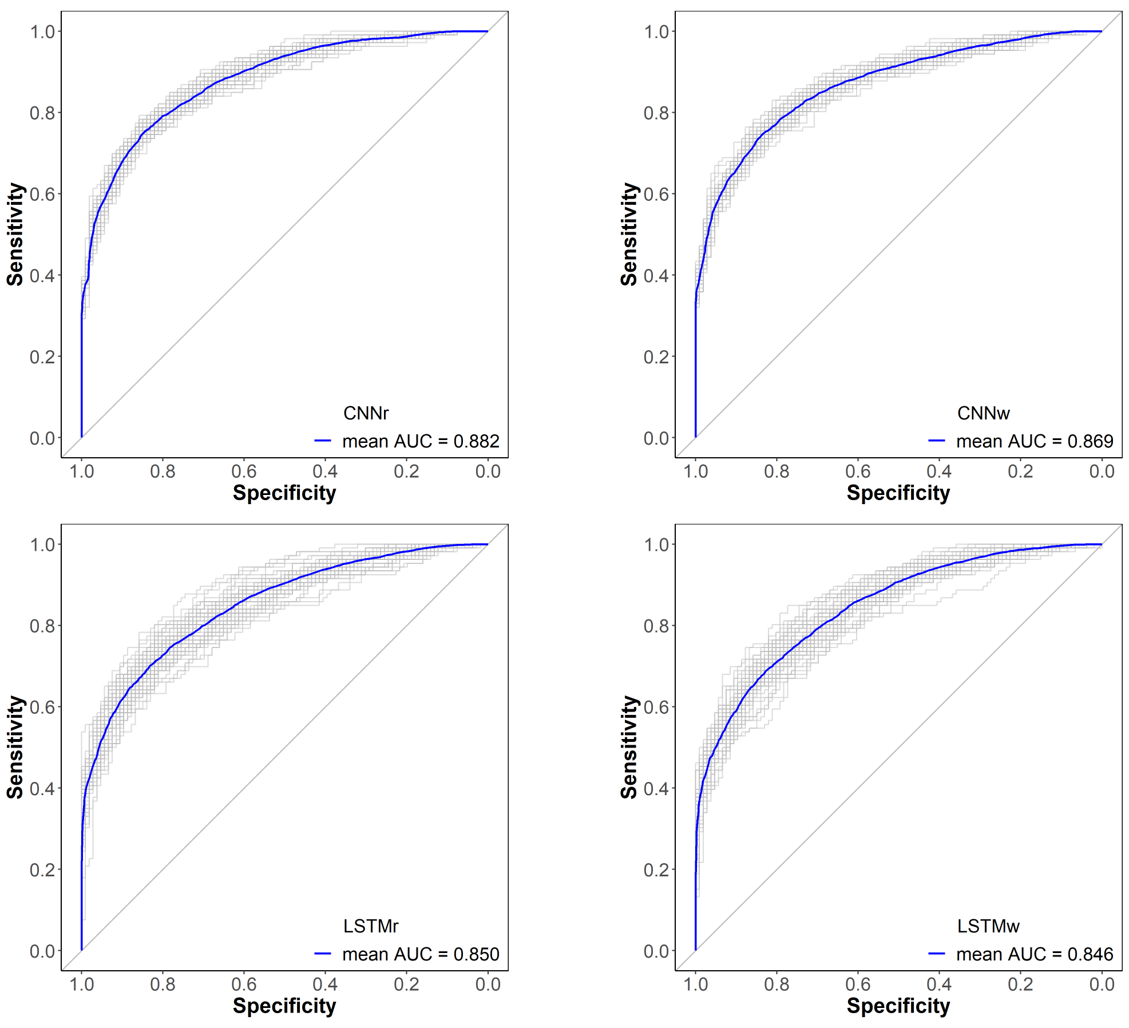

Supplement: Multimedia Appendix 3 [file medinform_v8i7e17784_app3.png]
